# FDG/PSMA discordance in [^177^Lu]Lu-PSMA radioligand therapy for mCRPC: a provisional target-sufficiency framework

**DOI:** 10.3389/fonc.2026.1935673

**Published:** 2026-08-25

**Authors:** Jialiang Deng, Guoxiong Ma, Weijin Chen, Songhao Wen, Nanhui Chen

**Affiliations:** 1 The First School of Clinical Medicine, Guangdong Medical University, Zhanjiang, Guangdong, China; 2 Meizhou People’s Hospital, Meizhou, Guangdong, China; 3 Affiliated Meizhou Hospital of Shantou University Medical College, Meizhou, China

**Keywords:** [^177^Lu]Lu-PSMA, dosimetry, FDG PET, FDG/PSMA discordance, metastatic castration-resistant prostate cancer, PSMA PET, radioligand therapy, target insufficiency

## Abstract

[^177^Lu]Lu-PSMA radioligand therapy (LuPSMA RLT) has improved outcomes for patients with metastatic castration-resistant prostate cancer (mCRPC), yet clinically meaningful response heterogeneity persists even among patients selected on the basis of PSMA PET positivity. A central limitation of patient-level PSMA eligibility is that it establishes target availability in at least part of the tumor burden, but it does not show whether all metabolically active lesions have sufficient PSMA expression, therapeutic retention, and delivered radiation dose. In this focused narrative review, we examine FDG/PSMA discordance as an imaging-suspected lesion-level phenotype of suspected target insufficiency rather than as a generic biomarker. We propose a provisional target-sufficiency framework that separates five research-testable states: concordant target-available disease, PSMA-positive target-density risk, focal suspected target-insufficient discordance, widespread suspected target-insufficient discordance, and delivery-insufficient target-positive disease. We synthesize dual-tracer eligibility studies, post-trial analyses of VISION- and TheraP-like criteria, FDG metabolic burden analyses, PSMA heterogeneity studies, post-therapy SPECT evidence, and LuPSMA dosimetry studies. Across predominantly retrospective cohorts, FDG-avid PSMA-low or PSMA-negative disease has been associated with adverse outcomes; however, this phenotype should not be conflated with high whole-body FDG metabolic tumor volume, low global PSMA uptake, measured absorbed-dose failure, or a proven resistance mechanism.

## 1 Introduction

PSMA-targeted radioligand therapy has become an important treatment option for metastatic castration-resistant prostate cancer (mCRPC). Early prospective studies established clinical activity and imaging predictors of response (1, 2). The VISION trial then established the survival benefit of [^177^Lu]Lu-PSMA-617 in patients selected by PSMA PET (3). Subsequent randomized, earlier-line, and real-world data have broadened the clinical context in which LuPSMA RLT is interpreted (4–6). Additional health-system-specific cohorts reinforce that such heterogeneity should be considered when trial-derived imaging criteria are extrapolated to routine practice (7). Yet PSMA-positive eligibility does not eliminate treatment failure. Some patients show primary nonresponse, early progression, mixed response, or progression within selected disease compartments despite meeting imaging selection criteria.

mCRPC is spatially and biologically heterogeneous: individual lesions may differ in PSMA expression, FDG avidity, proliferation rate, metastatic site, prior-treatment exposure, and therapeutic response. Quantitative VISION analyses and PSMA tumor-burden studies show that uptake intensity and target-positive volume are continuous risk markers rather than binary labels (8–10). Eligibility criteria and tracer choice also alter how PSMA-positive disease is operationalized (11–13). A PSMA PET-positive scan shows that part of the tumor burden presents an imageable target. It does not, however, guarantee that every metabolically active lesion has enough PSMA expression, ligand retention, or absorbed radiation dose to be controlled by PSMA-directed RLT. Lesion-level analyses also show heterogeneity by metastatic site (14).

This focus is deliberately narrower than a general review of LuPSMA biomarkers. Recent reviews have already summarized imaging and clinical biomarkers for PSMA-targeted RLT (15–17). A recent broad review places PSMA-targeted theranostics within the evolving integration of molecular imaging, targeted radionuclide therapy, and precision oncology (18). Management-oriented PSMA PET discussions and reviews of LuPSMA limitations provide more disease-specific context (19–24). Here, the question is different: how should FDG-avid PSMA-low or PSMA-negative disease be interpreted within otherwise PSMA-positive mCRPC, and how can that interpretation change candidate assessment, monitoring, and trial design?

The central distinction in this review is therefore between target availability and target sufficiency. Target availability refers to the presence of imageable PSMA-positive disease at patient or lesion level. Target sufficiency refers to the more demanding condition that metabolically active disease has adequate target expression and therapeutic delivery to be plausibly controlled by PSMA-directed RLT. FDG/PSMA discordance is best interpreted within this gap. FDG-high/PSMA-low or FDG-positive/PSMA-negative lesions represent metabolically active disease with insufficient visible PSMA target availability. In this review, that pattern is framed as an imaging-suspected lesion-level phenotype of suspected target insufficiency rather than as a fully validated mechanism of resistance.

Existing tools describe different parts of the problem. VISION-like criteria establish PSMA PET eligibility; TheraP-like selection adds FDG PET; PROMISE V2 and the Response Evaluation Criteria in PSMA PET/CT (RECIP) standardize reporting and response; and post-therapy SPECT or dosimetry assess delivery. None integrates baseline target density, focal or widespread discordance, and post-treatment delivery within a single lesion-level classification. The proposed framework connects these layers without replacing their established uses.

To reduce circularity, states should be assigned from prespecified imaging or delivery measurements, not subsequent treatment response. Existing studies provide no validated cutoff between focal and widespread discordance. Future protocols should therefore test lesion count, discordant FDG metabolic tumor volume fraction, organ distribution, and disease-compartment dominance as continuous variables before considering categorical thresholds. Here, focal discordance denotes a limited compartment within otherwise PSMA-positive disease; widespread discordance denotes broader, higher-burden, or clinically dominant disease. Target-density risk applies to PSMA-avid but quantitatively weak or heterogeneous disease, whereas delivery insufficiency requires post-treatment SPECT or absorbed-dose evidence.

Trial-derived imaging strategies make this problem clinically visible. VISION-like selection relies on PSMA PET to identify PSMA-positive disease and exclude protocol-defined PSMA-negative lesions (3). TheraP-like dual-tracer selection additionally uses FDG PET to detect metabolically active disease with low or absent PSMA expression (25). Post-trial and tracer-comparison studies show that these eligibility frameworks are not interchangeable (11–13, 26, 27). Post-trial eligibility analyses have shown that applying TheraP-like criteria to VISION-eligible patients can identify high-risk subgroups, with adverse outcomes often driven more strongly by FDG-avid discordant disease than by low PSMA uptake alone (26, 28). At the same time, end-stage cohorts caution against interpreting discordance as an automatic treatment prohibition, because some patients with few alternatives may still derive palliative or disease-control benefit (4, 26).

**Figure 1:**
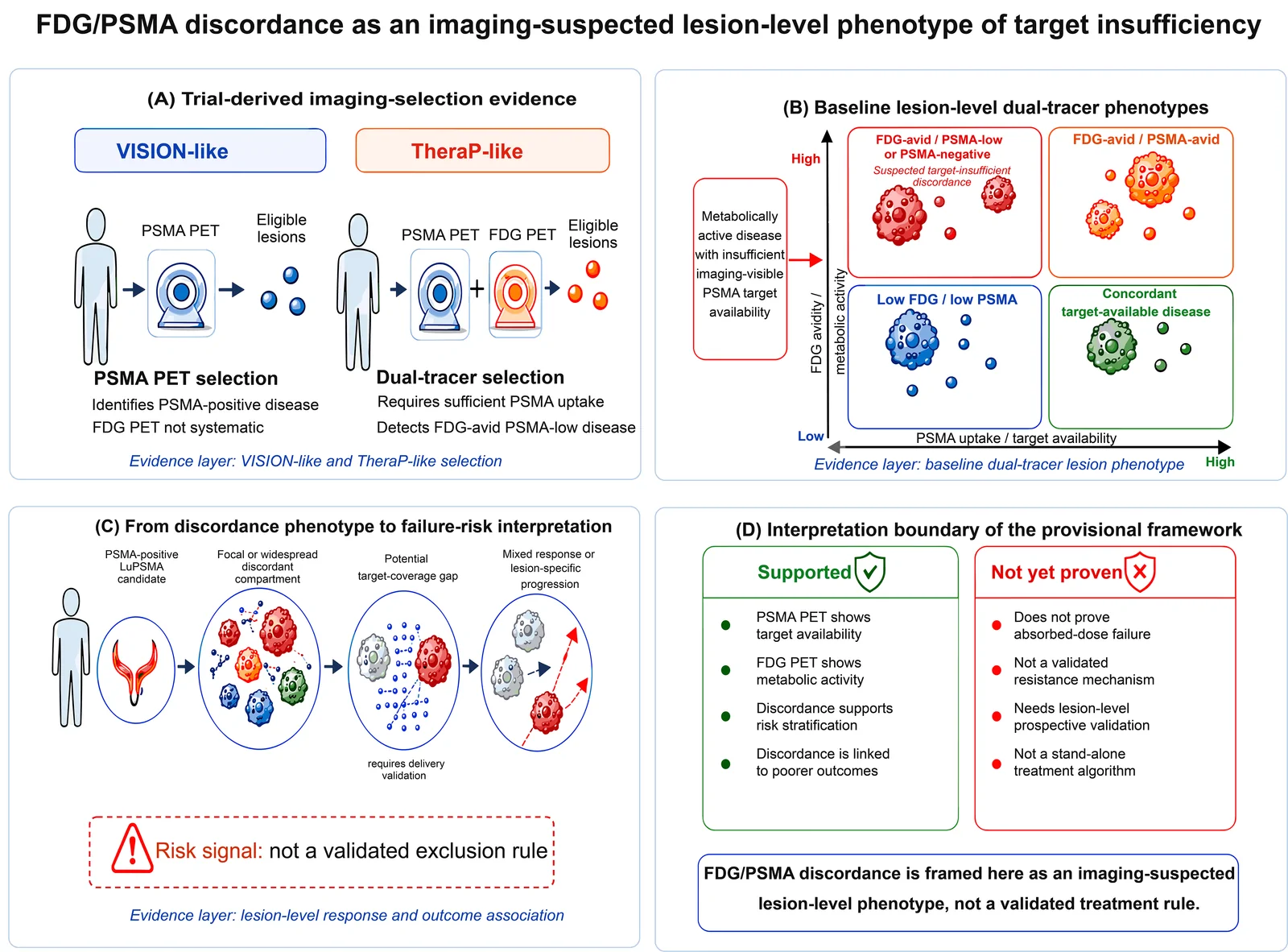
Provisional discordance-aware framework for imaging-suspected target insufficiency in metastatic castration-resistant prostate cancer. **(A)** VISION-like selection identifies PSMA-positive disease, whereas TheraP-like dual-tracer selection also detects FDG-avid, PSMA-low or PSMA-negative disease. **(B)** Baseline lesions are classified by FDG avidity and PSMA uptake; FDG-avid/PSMA-low or PSMA-negative lesions represent suspected target-insufficient discordance. **(C)** Focal or widespread discordance may indicate incomplete target coverage and a risk of mixed or lesion-specific progression, pending validation of therapeutic delivery and outcome. **(D)** Discordance supports risk stratification but does not establish absorbed-dose failure, a resistance mechanism, or a treatment-exclusion rule. Original synthesis by the authors. FDG, fluorodeoxyglucose; mCRPC, metastatic castration-resistant prostate cancer; PET, positron emission tomography; PSMA, prostate-specific membrane antigen.

**Figure 1** summarizes the scope of this review and introduces the same state logic as **Table 1**. PSMA PET establishes target availability, FDG PET identifies metabolically active disease, post-therapy SPECT or dosimetry tests therapeutic delivery, and lesion-level outcome determines whether the imaging-suspected state has clinical validity.

**Table 1:** Discordance-aware provisional target-sufficiency framework for PSMA-positive LuPSMA candidates.

Framework state	Imaging definition	Clinical question	Current use	Validation need	Candidate validation variables
Concordant target-available disease	PSMA-avid disease without known FDG-avid/PSMA-low or PSMA-negative lesions when FDG PET is available.	Is visible target adequate for standard LuPSMA selection?	Eligibility assessment and routine response monitoring	Confirm durable lesion control and no early delivery failure	PSMA-positive tumor volume; uptake intensity; absence of known FDG-avid/PSMA-low or PSMA-negative lesions when FDG PET is available.
PSMA-positive target-density risk	Low mean PSMA uptake, high PSMA-positive burden relative to uptake, organ-site risk, or heterogeneous uptake.	Is visible target quantitatively weak or heterogeneous?	Risk stratification; consider FDG PET when discordance is suspected	Prespecify uptake/burden thresholds linked to response, dosimetry, and lesion outcome	PSMA SUV_mean/SUV_max; PSMA-positive tumor volume; total lesion PSMA; uptake heterogeneity; organ-site distribution.
Focal suspected target-insufficient discordance	Limited FDG-avid/PSMA-low or PSMA-negative lesions within otherwise PSMA-positive disease.	Is a limited compartment outside PSMA target coverage?	Lesion tracking, structured reporting, multidisciplinary documentation, and trial stratification	Prospectively test lesion count, discordant-volume fraction, organ-site dominance, delivery, and lesion progression	Discordant lesion count; FDG uptake; lesion site; progression despite control elsewhere.
Widespread suspected target-insufficient discordance	High-burden or organ-dominant FDG-avid/PSMA-low or PSMA-negative disease constituting the dominant discordant compartment.	Is systemic disease largely outside PSMA target coverage?	Structured reporting, risk documentation, multidisciplinary review, and trial stratification; not an automatic exclusion rule	Validate discordant lesion count and FDG MTV fraction against survival and lesion-level failure	Discordant lesion count and FDG MTV; fraction of total FDG MTV; organ-site or disease-compartment dominance.
Delivery-insufficient target-positive disease	Baseline PSMA-positive lesions with weak cycle-1 SPECT uptake, low absorbed dose, or PET-to-dose discordance; requires post-therapy measurement.	Was visible target therapeutically under-delivered?	Research/adaptive-monitoring layer; not a stand-alone exclusion rule	Prospectively link baseline dual-tracer imaging, cycle-1 SPECT/dosimetry, and lesion outcome	Cycle-1 SPECT uptake; lesion absorbed dose; PET/SPECT mismatch; progression despite baseline PSMA uptake.

States are assigned from prespecified imaging or delivery measures, not treatment response. Baseline PSMA PET and, when available, FDG PET define target availability, target-density risk, and discordance; early post-therapy SPECT or dosimetry adds the delivery layer. States may coexist: target-density risk may accompany focal or widespread discordance, and delivery status overlays the baseline phenotype. Without FDG PET, discordance remains not assessed rather than concordant. Focal and widespread discordance are qualitative working labels; candidate variables should first be tested continuously and are not validated clinical thresholds.

### 1.1 Methods: focused narrative synthesis

This article is a focused, framework-building narrative review rather than a systematic review, scoping review, or meta-analysis. It organizes heterogeneous evidence on dual-tracer PET, PSMA heterogeneity, response assessment, post-therapy SPECT, and dosimetry around a provisional target-sufficiency framework. It does not claim exhaustive retrieval, formal evidence-completeness assessment, or pooled effect estimation.

PubMed and Web of Science were searched on June 24, 2026, using terms related to prostate cancer or metastatic castration-resistant prostate cancer, PSMA PET, radioligand therapy, lutetium-177, and theranostics. The searches identified 374 records in PubMed and 463 records in Web of Science. After conservative deduplication, 578 unique records remained. Title, abstract, and metadata screening identified 452 potentially relevant records. Following further relevance prioritization and removal of lower-priority or out-of-scope records, approximately 322 records were retained in the working literature library. This library was subsequently used for focused evidence assessment of FDG/PSMA discordance, PSMA heterogeneity, treatment-response assessment, post-therapy SPECT, and dosimetry.

Evidence directly addressing FDG/PSMA discordance, PSMA heterogeneity, treatment-response assessment, post-therapy SPECT, or dosimetry was then prioritized. Citation preference was given to pivotal trials, original clinical studies, reporting or consensus frameworks, and methodologically mature evidence syntheses. Small case series, case reports, studies of non-LuPSMA agents, and indirect biological studies were used only to address specific evidence gaps and were interpreted according to their methodological limitations. No formal risk-of-bias assessment or quantitative synthesis was performed. The complete PubMed and Web of Science search strategies are provided in **Supplementary Methods 1**.

## 2 Focal and widespread FDG/PSMA discordance as imaging-suspected target-insufficient states

Patient-level eligibility does not exclude lesion-level FDG/PSMA discordance. A patient may satisfy PSMA PET selection criteria while harboring either a limited FDG-avid PSMA-low lesion or a broader FDG-avid PSMA-negative compartment. These patterns correspond to the focal and widespread suspected target-insufficient states in **Table 1** and should not be collapsed into a single category. Focal discordance mainly raises lesion-level monitoring, local-control, and trial-stratification questions, whereas widespread discordance challenges the assumption that patient-level PSMA positivity equals systemic targetability (26–29).

**Figure 2:**
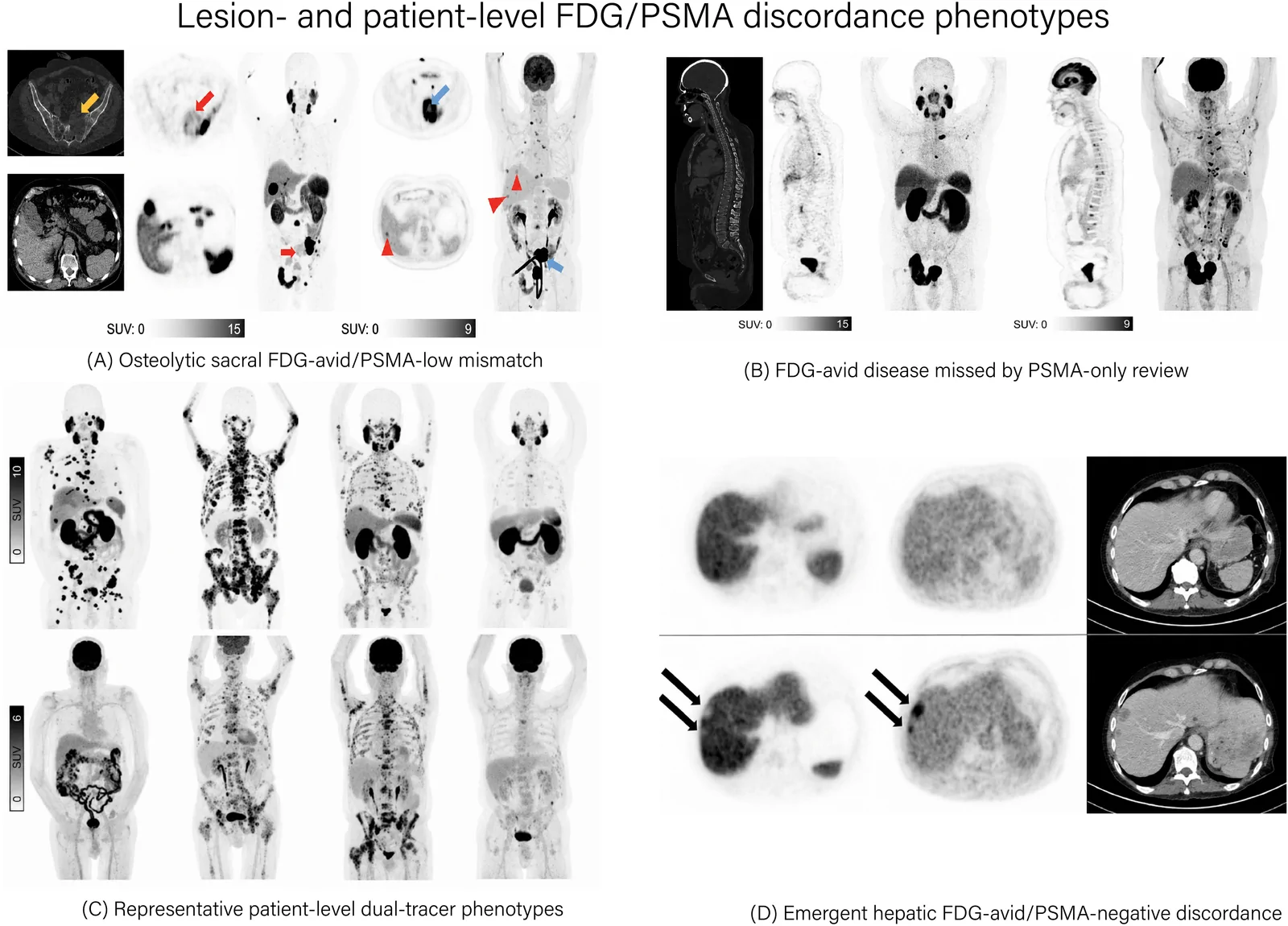
Lesion- and patient-level FDG/PSMA discordance phenotypes. **(A)** Osteolytic sacral disease with low PSMA uptake and intense FDG uptake; arrows identify the sacral lesion and additional FDG-avid liver lesions. **(B)** FDG-avid bone disease missed by a PSMA-only VISION-like review. **(C)** Patient-level dual-tracer phenotypes used to assess TheraP eligibility; each column represents a different patient. **(D)** New FDG-avid/PSMA-negative liver metastases after four cycles of PSMA-targeted radioligand therapy. These examples illustrate baseline and emergent discordance but do not establish absorbed-dose failure or a uniform resistance mechanism. **(A, B)** were adapted from material originally published in JNM: Seifert R, Telli T, Hadaschik B, Fendler WP, Kuo PH, Herrmann (K) Is 18F-FDG PET Needed to Assess ^177^Lu-PSMA Therapy Eligibility? A VISION-like, Single-Center Analysis. J Nucl Med. 2023;64:731-737. doi:10.2967/jnumed.122.264741. Original **Figures 2**, **3**. Copyright SNMMI. **(C)** was adapted from material originally published in JNM: Demirci RA, et al. PET-Based TheraP Eligibility and Outcomes of VISION-Eligible Patients with Metastatic Castration-Resistant Prostate Cancer Who Received ^177^Lu-PSMA-617: Importance of 18F-FDG-Avid Discordant Findings. J Nucl Med. 2025;66:47-53. doi:10.2967/jnumed.124.268167. Original **Figure 3**. Copyright SNMMI. **(D)** was adapted from Hartrampf PE, Lapa C, Serfling SE, Buck AK, Seitz AK, Meyer PT, Ruf J, Michalski (K) Development of Discordant Hypermetabolic Prostate Cancer Lesions in the Course of [^177^Lu]PSMA Radioligand Therapy and Their Possible Influence on Patient Outcome. Cancers. 2021;13:4270. doi:10.3390/cancers13174270. Original **Figure 3**. Copyright 2021 by the authors; distributed under the Creative Commons Attribution 4.0 International License (https://creativecommons.org/licenses/by/4.0/). Changes consisted of cropping, resizing, relabeling, and composite assembly. CT, computed tomography; FDG, fluorodeoxyglucose; PET, positron emission tomography; PSMA, prostate-specific membrane antigen.

In this review, lesion-level FDG/PSMA discordance is used as a working imaging category and refers to metabolically active disease on FDG PET with absent, low, or functionally inadequate visible PSMA uptake relative to surrounding target-positive disease. In **Table 1** terms, focal discordance denotes one or a few discordant lesions within otherwise target-available disease, while widespread discordance denotes a substantial discordant compartment. This distinction separates lesion-level mismatch from low global PSMA uptake, which belongs to PSMA-positive target-density risk, and from high whole-body FDG metabolic tumor volume, which reflects metabolic burden rather than mismatch by itself (**Figure 2**).

Reproducible state assignment requires prespecified acquisition and lesion-adjudication rules. PSMA and FDG PET should be performed within a defined interval, and studies should report tracer-specific uptake times, liver-reference criteria, and minimum measurable-lesion requirements; these variables should be documented rather than treated as universal thresholds (30). Because FDG uptake is not tumor-specific, inflammatory or infectious foci and possible second primary malignancies should be excluded through anatomic correlation and clinical review before discordance is assigned; Rosar et al. excluded characteristic inflammatory foci from their lesion-level analysis (31). Spatial correspondence should be confirmed through dual-tracer co-registration and expert review, with consensus adjudication for equivocal lesions (32). Radiotracer, scanner, reconstruction, and segmentation differences should also be reported because they may alter uptake measurements and state assignment.

The focal-discordance state is clinically important because PSMA-only selection can miss a small target-insufficient compartment. Seifert and colleagues evaluated patients with both PSMA and FDG PET and reported that a minority of patients had FDG/PSMA mismatch not captured by PSMA-only criteria (27). These data do not make FDG PET mandatory for every candidate, but they show why a limited discordant lesion should be recorded as a distinct state rather than diluted within patient-level eligibility.

The widespread-discordance state carries a different implication. Baseline FDG-positive/PSMA-negative lesions have been associated with adverse survival in patients undergoing PSMA-targeted RLT (29). In one frequently cited cohort, approximately one-third of treated patients had baseline FDG-positive/PSMA-negative lesions, and those patients had substantially shorter overall survival than patients without such lesions (29). These data support the adverse-risk meaning of discordance, but they do not prove measured absorbed-dose failure because lesion-level dosimetry and lesion-specific outcomes were not co-registered.

Post-trial analyses help place these states within trial-derived eligibility. In VISION-eligible patients treated with LuPSMA, TheraP-like ineligibility has been associated with poorer outcomes, and the adverse signal appears to be driven more strongly by FDG-avid discordant disease than by low PSMA uptake alone (28). This supports **Table 1** as a state-assignment framework: dual-tracer imaging can identify focal or widespread suspected target-insufficient disease that is not captured by PSMA PET selection alone.

Whole-body FDG metabolic tumor volume (MTV) remains relevant, but it should not be re-labeled as FDG/PSMA-discordant lesion volume. In the TheraP biomarker analysis, higher FDG MTV was associated with adverse outcomes and lower PSA response across treatment groups, whereas PSMA PET mean standardized uptake value (SUV_mean) appeared more closely associated with differential response to LuPSMA versus cabazitaxel (25). In a retrospective dual-tracer cohort, FDG-derived tumor volume was independently associated with overall survival across treatment groups. By contrast, PSMA PET SUV_mean was associated with a prostate-specific antigen decline of at least 50% (PSA50) among LuPSMA-treated patients, supporting distinct prognostic and response-related roles for the two tracers (30). In **Table 1** terms, FDG MTV modifies metabolic risk context, while focal or widespread mismatch identifies where PSMA-targeted therapy may fail to cover metabolically active disease.

Rosar and colleagues extended this distinction to lesion-level outcome in a retrospective single-center analysis of 267 FDG-avid metastases from 22 patients. After two LuPSMA cycles, 51 lesions (19.1%) progressed. Baseline PSMA maximum standardized uptake value (SUV_max) and the FDG-to-PSMA SUV_max quotient (FPQ), but not FDG SUV_max alone, discriminated lesion progression; the respective areas under the receiver operating characteristic curves (AUCs) were 0.89 and 0.90. An exploratory dual imaging progression prediction (DIPP) score achieved an AUC of 0.94. The proposed cutoffs (PSMA SUV_max <11.09 and FPQ ≥0.92) require external prospective validation and should not be treated as clinical thresholds because the study was retrospective, single-center, and did not include lesion dosimetry (31).

Discordance can also emerge during treatment. Hartrampf and colleagues described new FDG-positive/PSMA-negative lesions after LuPSMA treatment, with reported emergence after two and four treatment cycles in a subset of patients (33, 34). Such findings are best considered phenomenon-level and hypothesis-generating. Emergent discordance may reflect, or at least be compatible with, therapeutic selection pressure, unmasking of FDG-avid compartments after control of PSMA-avid disease, or ongoing clonal divergence. Current evidence is insufficient to treat emergent discordance as a validated mechanism of acquired resistance.

The validation implication is state-specific. Focal discordance requires lesion tracking to determine whether the discordant lesion progresses despite control elsewhere. Widespread discordance requires patient-level and lesion-level endpoints to determine whether the discordant compartment drives survival and radiographic failure. State assignment and response interpretation should be anchored to standardized PSMA PET reporting and response frameworks such as PROMISE V2, PSMA PET Progression (PPP) criteria, and RECIP (35–41). Quantitative lesion tracking may be supported by automated segmentation and tumor-burden assessment methods where validated (42–44). To test whether failure reflects inadequate therapeutic delivery, future studies should link baseline dual-tracer phenotypes to post-therapy SPECT uptake, absorbed-dose estimates, and lesion-specific outcomes (45–50). The evidence supporting these state distinctions is summarized in **Table 2**.

**Table 2:** Quantitative evidence map of dual-tracer phenotypes, target-density markers, and therapeutic-delivery measures.

Study/design	Sample	Imaging definition	Main findings	Analysis/adjustment	Key limitation	Reference
Buteau et al., 2022; randomized TheraP biomarker analysis	n = 200 (LuPSMA, 99; cabazitaxel, 101)	Prespecified PSMA PET SUV_mean ≥10 and FDG MTV ≥200 mL.	PSA50 with SUV_mean ≥10: 91% vs 47% (LuPSMA vs cabazitaxel); below 10: 52% vs 32%. High FDG MTV was associated with poorer outcomes across groups.	Randomized comparison; prespecified biomarker interaction analyses.	FDG MTV is a patient-level burden measure, not lesion-level discordance or dosimetry.	(25)
Seifert et al., 2023; retrospective single-center eligibility study	n = 89	Dual-tracer PET within 2 weeks; mismatch required FDG uptake above liver, PSMA uptake below reference tissue, and prespecified lesion size.	Mismatch: 16/89 (18%); agreement with PSMA-only VISION-like assessment, κ = 0.73; PSMA-only review missed 3/89 (3%).	Agreement analysis; no adjusted outcome model.	Retrospective eligibility study with heterogeneous tracers; no outcome or dose analysis.	(27)
Michalski et al., 2021; retrospective dual-tracer LuPSMA cohort	n = 54	≥1 baseline FDG-positive/PSMA-negative lesion.	Discordance: 18/54 (33%); median overall survival, approximately 6.0 vs 16.0 months; adjusted hazard ratio for death, 4.9 (95% CI, 1.7–14.3).	Multivariable Cox regression.	Small retrospective cohort; mixed tracers and no lesion-level dosimetry.	(29)
Demirci et al., 2025; retrospective VISION-eligible LuPSMA cohort	n = 75	TheraP criteria included PSMA SUV_max thresholds and excluded FDG-avid sites with inadequate PSMA uptake.	Thirty-one patients were TheraP-ineligible, including 24 with discordance. PSA50: 28% vs 67% (odds ratio, 0.19; 95% CI, 0.06–0.52); discordance was associated with overall survival (adjusted hazard ratio, 3.0; 95% CI, 1.3–6.8).	Multivariable logistic and time-to-event models with clinical/PET covariates.	Single-center retrospective cohort; cannot show that exclusion improves outcomes.	(28)
Telli et al., 2025; retrospective dual-tracer prognostic cohort	n = 152 (LuPSMA, 104; standard care, 48)	Visual mismatch plus quantitative FDG volume and PSMA SUV_mean.	Median overall survival with vs without mismatch: 4.5 vs 10.6 months. In the LuPSMA group, FDG volume was associated with overall survival (hazard ratio, 1.28; 95% CI, 1.02–1.61), and PSMA SUV_mean with PSA50 (odds ratio, 2.97; 95% CI, 1.27–8.16).	Multivariable Cox and logistic regression.	Nonrandom allocation; mismatch affected selection, and only two treated patients had mismatch.	(30)
Rosar et al., 2024; retrospective lesion-level dual-tracer study	22 patients; 267 lesions	Baseline PSMA SUV_max, FDG SUV_max, and FDG-to-PSMA quotient; progression after two cycles defined by increased FDG uptake.	Progression: 51/267 lesions. Areas under the curve: 0.89 for PSMA SUV_max, 0.90 for the FDG-to-PSMA quotient, and 0.94 for the combined model.	Receiver-operating-characteristic analysis; data-derived cutoffs.	Small single-center cohort; no external validation, explicit patient-level clustering, or dosimetry.	(31)
Hohberg et al., 2023; prospective imaging and dosimetry study	n = 30	Pretherapy lesion-to-kidney uptake ratio and serial SPECT dosimetry; response after three cycles.	Cycle-1 mean absorbed dose was higher in responding vs nonresponding lymph-node lesions (3.73 vs 1.86 Gy/GBq) and bone lesions (3.47 vs 1.48 Gy/GBq).	Primarily unadjusted group comparisons.	Small [^177^Lu]Lu-PSMA-I&T cohort without FDG PET; supports dose-response association, not discordance-mediated dose failure.	(48)
Ghodsi et al., 2026; retrospective cycle-1 SPECT prognostic study	n = 192	Cycle-1 24-h SPECT total-tumor SUV_mean quartiles versus baseline PSMA PET.	PET/SPECT SUV_mean correlation: r = 0.81; 41% changed quartile. Highest SPECT quartile: PSA50 odds ratio, 17.1; PSA progression-free survival hazard ratio, 0.17; overall survival hazard ratio, 0.42 (95% CI, 0.18–0.98).	Adjusted for baseline PET quartile and PET-to-SPECT interval.	Single-center; SPECT SUV_mean is a delivery surrogate, and the overall-survival signal was confined to the highest quartile.	(49)

This table presents selected core studies that directly inform the framework and is not intended as an exhaustive systematic evidence inventory.

## 3 PSMA heterogeneity and PSMA-positive target-density risk

PSMA-positive disease is not uniformly target-rich. The PSMA-positive target-density risk state in **Table 1** is distinct from FDG/PSMA mismatch: it describes disease that remains visibly PSMA-positive but is quantitatively weak, heterogeneous, high-burden, organ-site dependent, or biologically uneven. This distinction avoids a binary reading of PSMA PET; a patient may have detectable target expression yet still show insufficient or uneven target density for reliable disease control.

PSMA PET biomarkers are discussed here only insofar as they explain why patient-level eligibility incompletely captures treatment-failure risk and why lesion-aware interpretation is needed.

Whole-body PSMA metrics show why target availability is continuous rather than binary. PSMA-positive tumor volume, total lesion PSMA uptake, and average uptake intensity quantify different aspects of the target-positive compartment. In a bicentric cohort, PSMA total tumor volume, PSMA total lesion quotient (PSMA-TLQ; PSMA-positive tumor volume divided by SUV_mean), and related derived metrics integrating PSMA-positive tumor burden and uptake intensity were associated with overall survival after LuPSMA RLT (9). VISION quantitative PET analyses and organ-specific volumetric studies support a similar burden-to-intensity interpretation (8, 51). A meta-analysis of 23 studies further associated PSMA PET SUV_mean, tumor volume, and total-lesion PSMA with treatment outcomes, while emphasizing substantial methodological heterogeneity (52). These data support the target-density risk state, not FDG/PSMA discordance and not absorbed dose.

Measurement choices determine how the PSMA-positive target-density risk state is assigned. Quantitative VISION PET analyses and baseline semiquantitative PSMA PET studies support PSMA uptake intensity, particularly SUV_mean, and tumor-burden measures as continuous risk or response variables rather than binary eligibility labels (8, 10, 53). Visual scoring approaches may approximate quantitative SUV_mean for treatment-response prediction and could support pragmatic risk stratification when fully quantitative workflows are not available (54). However, estimates of PSMA-positive tumor burden depend on segmentation and thresholding choices, and automated or AI-based volumetric methods may improve reproducibility but require validation before replacing manual or semiautomated assessment (42–44). In a small dual-center study, PSMA SUV_mean within PSMA/FDG-concordant lesions was associated with PSA response, although the proposed quantitative threshold remains exploratory (32). None of these PSMA-side approaches can determine whether FDG-avid PSMA-low disease is present. For that reason, **Table 1** separates PSMA-positive target-density risk from focal or widespread suspected target-insufficient discordance.

Lesion-level and organ-specific heterogeneity further refine the target-density risk state. Baseline PSMA PET lesion-level and volumetric parameters have been associated with treatment response, but studies that did not include FDG PET did not directly assess FDG-positive/PSMA-negative discordance (55). Bone-dominant and bone-only CRPC cohorts illustrate why skeletal distribution should be treated as a distinct context when interpreting PSMA PET burden and lesion-level response (51, 56). These findings support target-density risk and organ-site risk, while remaining indirect evidence for discordance.

Molecular response patterns after PSMA-targeted radionuclide therapy further illustrate spatial heterogeneity. In an analysis of patients treated with [225Ac]Ac-J591, lesion response rates differed by metastatic site (14). This alpha-emitter antibody study is not direct evidence for [^177^Lu]Lu-PSMA-617 and did not include FDG PET or lesion dosimetry. It is best viewed as indirect, concept-generating evidence that patient-level PSMA positivity can mask lesion-level response heterogeneity.

Tissue-level evidence reinforces the same caution. PET uptake is a clinically powerful readout, but it is not a direct microscopic map of PSMA protein expression. Spatial resolution, partial-volume effects, tracer biology, necrosis, prior therapy, and sampling differences can separate PET-level uptake from tissue-level expression. Laarhuis and colleagues reported that PSMA PET uptake at biopsied lesions did not correlate strongly with PSMA immunohistochemistry in a small mCRPC cohort undergoing PSMA-targeted RLT, and that low PSMA expression or high proliferative markers were associated with poorer outcomes (57). These findings should be interpreted carefully because biopsy samples are focal and subgroups were small, but they support the premise that PET positivity does not guarantee homogeneous microscopic target expression. PSMA heterogeneity spans cellular, spatial, lineage, organ-site, and temporal levels, with lineage plasticity and altered FOLH1 regulation providing plausible biological contexts for PSMA-low disease (58).

Radiomics and texture studies suggest that PSMA heterogeneity can be quantified, but they remain exploratory. PSMA PET texture features have been associated with PSA change or PSA50 response in small LuPSMA cohorts (59, 60). These studies do not prove that heterogeneity always represents resistant or PSMA-negative disease. Heterogeneous but intense PSMA uptake within targetable disease is different from FDG-avid/PSMA-low discordance. A mature framework should therefore distinguish PSMA uptake heterogeneity, microscopic PSMA expression heterogeneity, FDG/PSMA discordance, and delivered dose heterogeneity rather than treating them as interchangeable.

Standardized reporting is a prerequisite for comparing these phenomena. PROMISE V2 and PSMA PET reporting guidance provide structured language for describing disease distribution, PSMA expression, PSMA-positive tumor volume, and response assessment on the PSMA side of the equation (35, 36). However, response and progression classifications still vary across PPP, RECIP, RECIST-based, PCWG-adapted, PERCIST-based, and PET-specific frameworks, and the same patient may be categorized differently depending on the endpoint definition used (37–39, 61). Reviews focused on PSMA PET response assessment further emphasize that PSA kinetics, conventional imaging, PSMA PET progression, new lesions, and quantitative tumor-volume changes should not be treated as interchangeable endpoints (35, 40, 62). Early and interim response studies after LuPSMA RLT support this caution, showing that post-treatment PSMA PET parameters, total tumor volume changes, and early biochemical or radiographic responses require interpretation in clinical context (63–66). RECIP-based studies also suggest that PSMA PET response frameworks may provide prognostic information, but their performance can depend on tracer, timing, and endpoint construction (37, 38, 41, 65). These frameworks do not define FDG/PSMA discordance, but they standardize the PSMA-positive disease compartment. Without consistent PSMA reporting and endpoint definitions, the incremental value of FDG PET becomes difficult to evaluate.

In **Table 1** terms, PSMA heterogeneity explains why target availability can be quantitatively insufficient, but it is not the final explanation for discordant failure. It supports the PSMA-positive target-density risk state and provides biological plausibility for target-insufficient compartments; only dual-tracer imaging, delivery assessment, and lesion-level follow-up can determine which state is clinically decisive.

## 4 Therapeutic delivery, dosimetry, and delivery-insufficient target-positive disease

Baseline PSMA positivity does not guarantee adequate therapeutic delivery. The delivery-insufficient target-positive state in **Table 1** describes lesions that are visible on baseline PSMA PET but show weak post-therapy uptake or low absorbed dose. Diagnostic PSMA uptake and absorbed dose are therefore not interchangeable. Pretherapy PET-to-dose studies show that baseline uptake only imperfectly predicts subsequent delivered radiation (45). Tumor-to-organ ratios, post-therapy SPECT distribution, and lesion absorbed dose capture delivery more directly (48, 49). Tumor sink and dose-effect studies further show that radioligand distribution varies across patients and lesions (67–70).

**Figure 3:**
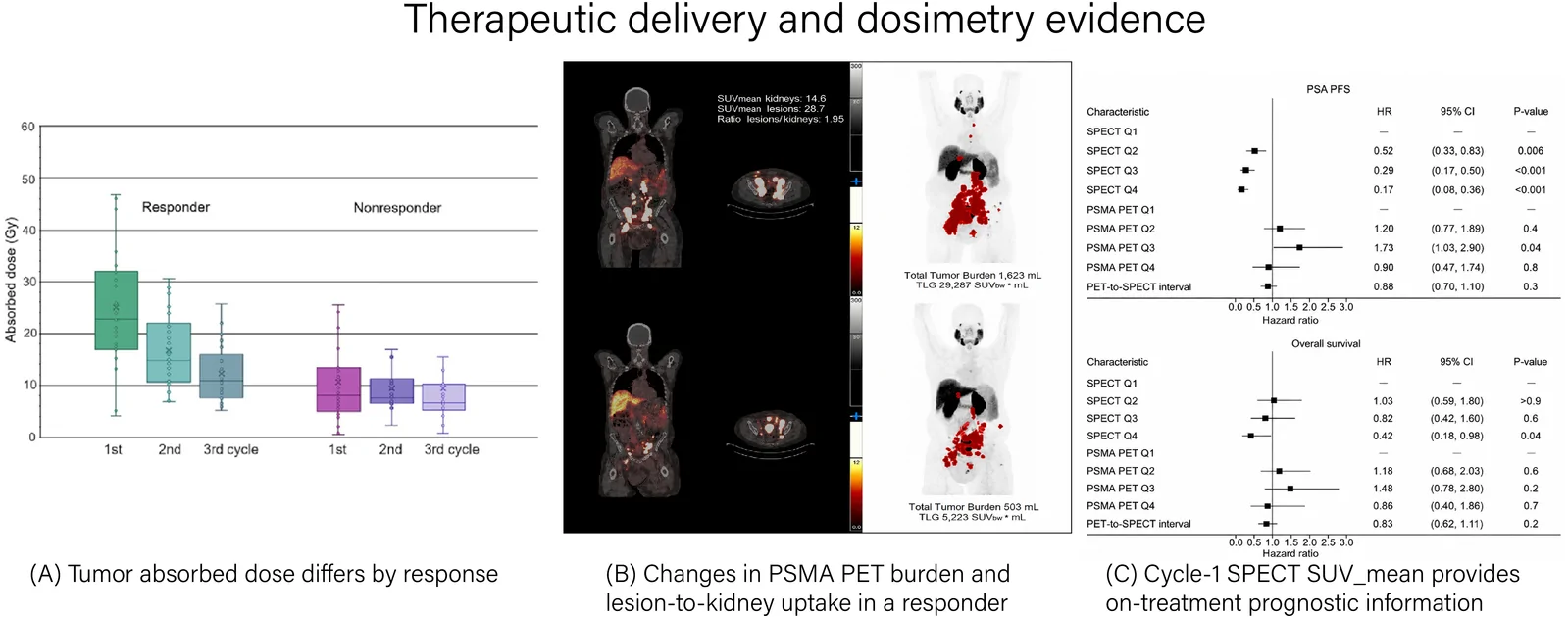
Therapeutic delivery and dosimetry evidence. **(A)** Mean absorbed dose to bone lesions across three cycles of [^177^Lu]Lu-PSMA-I&T in responders and nonresponders. **(B)** A responder before cycle 1 and after cycle 3, showing changes in PSMA PET tumor burden and the lesion-to-kidney uptake ratio. **(C)** Adjusted associations of cycle-1 SPECT SUV_mean quartiles with PSA progression-free and overall survival after [^177^Lu]Lu-PSMA-617. These findings support dose-response and on-treatment prognostic interpretations but do not establish absorbed-dose failure in FDG/PSMA-discordant lesions because FDG PET, lesion dosimetry, and lesion-specific outcomes were not co-registered. **(A, B)** were adapted from material originally published in JNM: Hohberg M, Reifegerst M, Drzezga A, Wild M, Schmidt M Prediction of Response to ^177^Lu-PSMA Therapy Based on Tumor-to-Kidney Ratio on Pretherapeutic PSMA PET/CT and Posttherapeutic Tumor-Dose Evaluation in mCRPC. J Nucl Med. 2023;64:1758-1764. doi:10.2967/jnumed.122.264953. Original **Figures 2**, **3**. Copyright SNMMI. **(C)** was adapted from material originally published in JNM: Ghodsi A, Demirci RA, Gulati R, et al. Prognostic Value of Cycle 1 SPECT SUV_mean in Patients with Metastatic Castration-Resistant Prostate Cancer Treated with ^177^Lu-PSMA-617. J Nucl Med. 2026;67:921-925. doi:10.2967/jnumed.125.271593. Figure 4 in the cited source article. Copyright SNMMI. Changes consisted of cropping, resizing, relabeling, and composite assembly. CI, confidence interval; FDG, fluorodeoxyglucose; HR, hazard ratio; OS, overall survival; PET, positron emission tomography; PSA-PFS, prostate-specific antigen progression-free survival; PSMA, prostate-specific membrane antigen; SPECT, single-photon emission computed tomography; SUV_mean, mean standardized uptake value.

The delivery-insufficient state sits downstream of target availability. Diagnostic PSMA uptake reflects target expression, tracer pharmacokinetics, lesion size, background activity, image timing, reconstruction, and partial-volume effects. Therapeutic retention depends on administered ligand, receptor binding and internalization, clearance, lesion perfusion, and tumor microenvironment. Absorbed dose adds time-integrated activity, lesion mass, spatial dose distribution, and radiobiologic sensitivity. **Figure 3** summarizes this chain using post-therapy imaging and dosimetry evidence blocks.

Peters and colleagues illustrate the problem directly. In a pilot dosimetry study using pretherapeutic PSMA PET and post-therapy SPECT-derived absorbed dose, organ-level absorbed dose prediction was more robust than lesion-level prediction, and lesion SUV_max did not significantly correlate with SPECT-derived absorbed dose (45). Although this was a small hormone-sensitive prostate cancer cohort rather than direct mCRPC resistance evidence, it provides an important methodological caution: SUV alone should not be used as a surrogate for delivered therapeutic radiation.

Systematic dosimetry evidence reinforces the same point. A systematic review and meta-analysis of LuPSMA dosimetry studies found substantial variability in reported organ and tumor absorbed doses across protocols, ligands, lesion types, segmentation methods, reconstruction approaches, and partial-volume corrections (46). These data support delivery heterogeneity, not a fixed universal resistance threshold. Cohort-average absorbed dose should not be overinterpreted as a patient-level or lesion-level decision rule.

Comparative dosimetry studies also argue against relying on administered activity alone. Schuchardt and colleagues compared [^177^Lu]Lu-PSMA-I&T and [^177^Lu]Lu-PSMA-617 under a common dosimetry protocol and reported broadly comparable mean tumor absorbed doses but substantial variability between patients and lesions (47). This supports further evaluation of patient-based dosimetry as a tool for more individualized LuPSMA RLT, particularly when failure is suspected to arise from heterogeneous target expression or incomplete target coverage.

Lesion-level dose-response evidence provides the strongest bridge between PSMA heterogeneity and treatment failure. Hohberg and colleagues evaluated pretherapeutic PSMA PET lesion-to-kidney uptake ratios, post-therapy SPECT/CT-derived absorbed dose, and PSA response after [^177^Lu]Lu-PSMA-I&T in mCRPC. Responders and nonresponders had similar administered activity and kidney absorbed dose, but lesion absorbed dose differed substantially, especially in bone and nodal lesions (48). This supports a tumor-specific delivery interpretation of nonresponse: failure may occur even when organ exposure is acceptable, if tumor lesions receive insufficient dose.

This dose-response evidence should be interpreted with ligand- and design-specific caution. Hohberg and colleagues evaluated [^177^Lu]Lu-PSMA-I&T rather than [^177^Lu]Lu-PSMA-617 and did not include baseline FDG PET co-registration; therefore, the study supports a delivery-based interpretation of nonresponse but cannot prove absorbed-dose failure in FDG-positive/PSMA-negative lesions.

This delivery state also clarifies the relationship between dosimetry and FDG/PSMA discordance. A truly PSMA-negative FDG-avid lesion would be expected to receive limited PSMA-targeted dose, whereas a PSMA-low lesion may occupy an intermediate zone of incomplete target coverage. However, studies without FDG PET cannot assign discordance, and studies without dosimetry cannot assign delivery insufficiency. The framework therefore keeps target-insufficient discordance and delivery-insufficient target-positive disease as related but distinct states.

Post-therapy SPECT can reveal therapeutic retention beyond baseline PET. Baseline PSMA PET and cycle-1 [^177^Lu]Lu-PSMA SPECT are related, but not interchangeable. PET-to-dose studies show limits of pretreatment PET prediction (45, 48). Case-level PET/SPECT/FDG discordance illustrates why these imaging layers may diverge in individual patients (50). Ghodsi and colleagues reported a strong correlation between pretreatment PSMA PET SUV_mean and cycle-1 SPECT SUV_mean, although a substantial proportion of patients changed uptake quartile between modalities. In adjusted analyses, higher cycle-1 SPECT SUV_mean quartiles were independently associated with PSA50 response and PSA progression-free survival (PSA-PFS) (49). This supports on-treatment SPECT as an early delivery surrogate, but SPECT SUV_mean is not complete absorbed-dose dosimetry.

Adaptive dosimetry-guided treatment is an emerging extension of delivery assessment. In prospective protocols, serial quantitative SPECT/CT or absorbed-dose estimates could inform subsequent-cycle activity or timing according to tumor delivery, cumulative normal-organ exposure, and interim response. Interpatient dose heterogeneity and the prognostic information from cycle-1 SPECT support further evaluation of this approach (46, 49). Tumor-sink studies also suggest that biodistribution may change as PSMA-positive tumor burden declines (69). These observations do not establish validated dose thresholds or show that adaptive adjustment improves clinical outcomes; quantitative SPECT uptake also should not be treated as complete absorbed-dose dosimetry. Adaptive strategies therefore remain investigational.

The missing dataset is now defined by **Table 1**: each lesion should be assigned a baseline phenotype, tested for early therapeutic delivery, and followed for lesion-specific outcome. The decisive dataset would co-register baseline FDG PET, baseline PSMA PET, early post-therapy SPECT or dosimetry, and lesion-specific progression within the same lesions.

## 5 Discordance-aware risk stratification and future validation

The five states define distinct validation questions rather than a clinical eligibility algorithm. Validation should proceed in stages: assign the baseline PSMA/FDG phenotype, quantify PSMA target density, assess therapeutic delivery, and then link each state to lesion-level and patient-level outcomes.

The framework complements VISION-like PSMA screening, TheraP-like dual-tracer assessment, RECIP-based response evaluation, and post-therapy SPECT or dosimetry. Its present contribution is to connect each tool to a defined failure phenotype and validation endpoint; incremental predictive or decision-making value remains to be demonstrated (**Table 3**).

**Table 3:** Existing tools versus the provisional five-state framework.

Existing tool	Current role	Framework contribution	Not established	Potential gap without framework
PROMISE V2/PSMA PET reporting	Standardizes PSMA PET extent, expression, and response reporting	Anchors PSMA-side target availability and target-density risk before FDG/PSMA interpretation	Does not define FDG/PSMA discordance or delivery insufficiency	FDG/PSMA mismatch remains outside PSMA-only reporting.
VISION-like PSMA PET eligibility	Identifies protocol-defined PSMA-positive disease	Separates patient-level target availability from lesion-level target sufficiency	Does not replace VISION eligibility or define a treatment indication	Patient-level positivity may be mistaken for lesion-level sufficiency; FDG-avid/PSMA-low disease may be missed.
TheraP-like dual-tracer selection	Detects FDG-avid/PSMA-low or PSMA-negative disease	Separates focal from widespread suspected target-insufficient discordance	Does not define a universal exclusion rule	Binary exclusion may obscure focal versus widespread discordance.
RECIP/PSMA PET response criteria	Standardizes PSMA PET response/progression	Links response patterns to baseline state and lesion-level failure	Does not establish progression mechanism	Post-treatment response is classified without a baseline failure phenotype.
Post-therapy SPECT/dosimetry	Assesses delivery or absorbed dose more directly than diagnostic PET	Defines delivery-insufficient target-positive disease as a separate layer	Does not show that low absorbed dose causes all discordant-lesion failures	Delivery may be measured without linkage to the baseline dual-tracer phenotype.
Current clinical workflow	Supports eligibility, selection, response, and delivery assessment	Integrates these steps into a lesion-level validation framework	Has no demonstrated incremental predictive or decision value	Target availability, density risk, discordance extent, and delivery insufficiency may remain conflated.

Post-trial analyses applying TheraP-style dual-tracer criteria to VISION-eligible cohorts identify subgroups with poorer outcomes, with the adverse signal driven more by FDG-avid/PSMA-low or PSMA-negative lesions than by low PSMA uptake alone (28).

A worked comparison illustrates this difference. Consider a patient who meets VISION-like PSMA criteria but has one FDG-avid, PSMA-negative liver lesion. A PSMA-only approach would classify the patient as eligible, whereas strict TheraP-like criteria might not. RECIP is not applicable before treatment, and dosimetry cannot characterize therapeutic delivery until radioligand administration. The framework would record focal suspected target-insufficient discordance, prompting prespecified lesion tracking, explicit trial stratification, and careful interpretation of mixed response. By contrast, diffuse low PSMA uptake without an FDG-avid, PSMA-negative lesion would indicate PSMA-positive target-density risk. Adequate baseline PSMA uptake followed by low cycle-1 SPECT uptake or low absorbed dose would indicate delivery-insufficient target-positive disease. These distinctions do not create a new clinical rule; they prevent biologically and methodologically different failure patterns from being collapsed into one label.

Validation questions differ by state. Concordant disease requires confirmation of durable lesion control under standard selection. Target-density risk should be linked to prespecified uptake and burden measures; focal discordance to lesion-specific progression despite control elsewhere; widespread discordance to radiographic and survival outcomes; and delivery insufficiency to early SPECT or absorbed-dose measures associated with lesion control.

Prospective validation should proceed in three stages. First, a multicenter observational registry should assess feasibility and reproducibility using prespecified dual-tracer acquisition, lesion adjudication, and spatial co-registration of baseline PET with cycle-1 SPECT or dosimetry. Focal discordant lesions should be tracked individually, whereas widespread discordance should initially be analyzed as a continuous burden measure. Second, an independent prognostic-validation cohort should relate baseline phenotypes and early delivery measures to lesion control, mixed response, radiographic progression-free survival, and overall survival. Analyses should account for lesions clustered within patients and compare conventional models with models that add the proposed framework; calibration, discrimination, and clinical net benefit would test incremental value. Only after reproducibility and incremental performance have been demonstrated should pragmatic randomized trials evaluate state-informed monitoring or protocolized treatment adaptation against standard care. Sample-size planning should be event-driven and account for phenotype prevalence and within-patient clustering. Until then, the framework should not be used to withhold standard LuPSMA RLT.

**Table 4** aligns each state with its evidence maturity, minimum imaging requirements, preferred endpoints, and interpretive limits. Evidence is strongest for clinical associations and delivery heterogeneity; the lesion-level chain linking baseline phenotype, delivered dose, and outcome remains incomplete.

**Table 4:** State-specific validation roadmap for the provisional target-sufficiency framework.

Framework state	Evidence	Required imaging	Validation	Endpoints	Boundary	References
Concordant target-available disease	Trial-based PSMA-selection evidence	Baseline PSMA PET; no known clinically relevant FDG-avid/PSMA-low disease if FDG PET is available	Confirm lesion control under standard LuPSMA selection	PSA50, rPFS, OS, lesion-level control	Concordance does not guarantee adequate dose to every lesion	(3, 8, 25)
PSMA-positive target-density risk	Quantitative PSMA PET evidence	PSMA uptake, tumor volume, burden, distribution, and heterogeneity	Link prespecified uptake/burden thresholds to response, dose, and lesion outcome	PSA50, rPFS, OS, lesion absorbed dose, lesion progression	Low uptake or high burden is not FDG/PSMA discordance	(8–10, 42–44)
Focal suspected target-insufficient discordance	Dual-tracer clinical and exploratory lesion-level evidence	One/few FDG-avid/PSMA-low or -negative lesions within otherwise PSMA-positive disease	Co-register baseline PET, post-therapy SPECT/dosimetry, and lesion progression	Lesion-specific progression, oligoprogression, mixed response, local failure	Focal discordance alone should not exclude LuPSMA RLT	(26–29, 31)
Widespread suspected target-insufficient discordance	*Post hoc* eligibility/adverse-risk evidence	Multiple or high-burden discordant lesions, distinct from total FDG MTV	Test standardized discordance-burden measures against alternative strategies	rPFS, OS, symptomatic and lesion-specific progression	Adverse-risk phenotype, not a universal exclusion rule	(25, 26, 28, 29)
Delivery-insufficient target-positive disease	LuPSMA SPECT/dosimetry evidence; limited lesion-level validation	Baseline PSMA-positive lesions with low cycle-1 SPECT uptake, absorbed dose, or PET-to-dose discordance	Link early SPECT/dosimetry to lesion control and baseline phenotype	Absorbed dose, SPECT uptake, lesion control, PSA-PFS	Diagnostic PSMA SUV is not absorbed dose	(45–50)
Cross-state reporting and endpoint discipline	Reporting/standardization evidence	PROMISE V2, PPP, RECIP, RECIST/PCWG, FDG definitions, and lesion tracking	Harmonize state assignment, endpoint hierarchy, and subgroup analyses	Patient-level and lesion-level composite endpoints	Standardization does not replace prospective validation	(35–41, 61, 65)

## 6 Discussion

This review separates target availability, target-density risk, discordance burden, and therapeutic delivery within an outcome-independent framework. It links each failure phenotype to the evidence needed for validation instead of treating these features as parallel biomarkers.

Most available studies are retrospective and lack lesion-level co-registration of baseline dual-tracer PET, post-therapy SPECT uptake, absorbed-dose estimates, and lesion-specific outcomes. For now, FDG/PSMA discordance should be treated as an imaging-suspected phenotype of target insufficiency rather than a proven mechanism of resistance. Prospective trials that predefine imaging acquisition, co-register baseline and post-therapy imaging, and report lesion-level dosimetry and outcomes are needed to determine whether discordant lesions fail because of absent target expression, inadequate ligand retention, or subtherapeutic absorbed dose.

Boundary of practice. Because the five-state framework is provisional, its current use should be limited to research stratification, structured imaging interpretation, multidisciplinary discussion, lesion-level follow-up planning, and prospective trial design. Acceptable uses include explicitly reporting FDG/PSMA discordant lesions, tracking discordant lesions on follow-up imaging, stratifying patients in clinical trials, and using early SPECT or dosimetry to study delivery-insufficient target-positive disease. Unacceptable uses include denying standard LuPSMA RLT solely on the basis of a discordance label, treating widespread discordance as an absolute exclusion criterion, using focal discordance as an independent indication for local ablative therapy, or presenting the framework as a validated treatment-selection algorithm.

For clinicians, the provisional target-sufficiency framework encourages lesion-aware reporting and selective FDG PET when focal discordance would change management. Until prospective lesion-level dosimetry data are available, discordance should prompt closer monitoring and multidisciplinary discussion rather than an absolute contraindication to [^177^Lu]Lu-PSMA RLT.

For researchers, the framework defines testable imaging states and endpoints. Trials should predefine dual-tracer acquisition, co-register baseline PET with post-therapy SPECT or dosimetry, and report lesion-specific outcomes to clarify whether discordant lesions fail because of absent target expression, delivery insufficiency, or biological resistance.

### 6.1 Alternative mechanisms for discordance-associated treatment failure

A serious alternative interpretation is that FDG/PSMA discordance is not a causal pathway to LuPSMA failure, but an epiphenomenon of aggressive biology. Under this model, discordant lesions have poor outcomes because they mark dedifferentiated, metabolically active, treatment-refractory disease; loss or reduction of visible PSMA is one feature of that state rather than the primary mechanism of resistance. This possibility is consistent with the current evidence base because most clinical studies link discordance to outcome without simultaneously measuring lesion-level genomics, microenvironment, absorbed dose, and lesion-specific progression.

Several non-mutually exclusive mechanisms should therefore be considered. First, neuroendocrine transdifferentiation or lineage plasticity can produce PSMA-low or PSMA-negative disease with high metabolic activity, making FDG-positive/PSMA-low lesions a marker of dedifferentiated biology rather than only a delivery problem (71–73). Second, adverse genomic programs, including TP53/RB1 loss and PI3K/PTEN pathway alterations often discussed in aggressive or treatment-refractory prostate cancer, could drive dedifferentiation and resistance to systemic therapy independently of PSMA-targeted radioligand delivery; this mechanism requires molecular confirmation rather than inference from imaging alone (72–74). Third, hypoxia-linked metabolic adaptation, lactate-rich tumor microenvironments, and other microenvironmental factors may increase glycolytic FDG uptake and alter radiosensitivity without necessarily changing PSMA expression in a simple binary way (75). Fourth, clonal evolution under treatment pressure may reveal or select metabolically active target-low subclones during or after LuPSMA RLT, producing emergent discordance or mixed response (34, 72, 73).

These alternatives shift discordance from a presumed delivery mechanism to a risk phenotype with several plausible causes. Distinguishing among them will require lesion-matched imaging, molecular, and outcome data.

## 7 Limitations

This framework-building narrative synthesis is limited by heterogeneity across imaging protocols, eligibility criteria, tracers, response definitions, treatment lines, and endpoint timing. The absence of formal risk-of-bias assessment and pooled analysis further limits the strength of cross-study comparisons.

Second, FDG/PSMA discordance is not uniformly defined. Studies vary in whether discordance is assessed visually or quantitatively, whether thresholds are lesion-based or patient-based, whether PSMA-low and PSMA-negative lesions are grouped, and whether discordance is measured at baseline or during treatment.

Focal and widespread discordance remain qualitative working labels. No natural cutoff currently separates these states, and the framework does not assume that such a cutoff necessarily exists. Future studies need to test continuous variables such as discordant lesion number, discordant FDG metabolic tumor volume, the proportion of discordant FDG MTV relative to total FDG MTV, organ-site dominance, and whether discordant disease represents the dominant disease compartment. Only reproducible nonlinear risk patterns would justify categorical cut points. Until these variables are prospectively validated, the label “widespread discordance” must not be used to deny LuPSMA RLT outside a clinical trial.

Third, the most important mechanistic link remains incomplete. Few studies co-register baseline FDG PET, baseline PSMA PET, post-therapy SPECT or dosimetry, and lesion-specific outcome within the same lesions. Therefore, discordance should not be equated with measured absorbed-dose failure.

Fourth, several evidence sources should be interpreted cautiously because they are small, retrospective, single-center, treatment-line specific, or based on special populations such as end-stage mCRPC, hormone-sensitive pilot cohorts, non-LuPSMA agents, or exploratory radiomics analyses. Emerging studies using AI-based PSMA burden analysis, bone-dominant volumetric assessment, or early post-therapy SPECT require independent validation before their findings can be generalized.

## 8 Future perspectives

Future studies should use prospective, lesion-matched datasets with standardized discordance definitions and prespecified outcomes. Integrating dual-tracer PET with early delivery measures would allow each state to be tested against subsequent lesion control.

Prospective trials can use discordance states for stratification or predefined subgroup analyses. Any state-guided adaptation should be tested against standard selection in a protocolized setting.

Finally, future frameworks should integrate biology and delivery rather than choosing between them. PSMA heterogeneity, metabolic aggressiveness, radioligand retention, absorbed dose, and tumor radiosensitivity may all contribute to treatment failure. A clinically useful model will need to account for how these layers interact at lesion level.

Applicability to earlier treatment lines and more complex therapeutic settings requires dedicated validation rather than assumed generalization.

## 9 Conclusion

FDG/PSMA discordance identifies metabolically active disease that may not be adequately represented by PSMA PET in otherwise eligible mCRPC. The proposed framework separates this phenotype from target-density risk and post-treatment delivery insufficiency, providing a common structure for lesion tracking, trial stratification, and validation. Its clinical value now depends on prospective lesion-matched imaging and outcome data. Until those data are available, the framework should be used for research classification, not individual treatment decisions.
