# Supplementary material for: FDG/PSMA discordance in [177Lu]Lu-PSMA radioligand therapy for mCRPC: a provisional target-sufficiency framework
Source: Front Oncol. 2026 Aug 25;16:1935673. doi: 10.3389/fonc.2026.1935673 (PMC13551417; doi:10.3389/fonc.2026.1935673)
Supplement: Supplementary file 1 [file SupplementaryFile1.docx]

**Supplementary Methods 1. Literature Search Strategies**

Database searches were conducted on June 24, 2026. The core strategies were designed to retrieve literature at the intersection of prostate cancer, PSMA PET, and radioligand therapy. FDG/PSMA discordance, PSMA heterogeneity, response assessment, post-therapy SPECT, and dosimetry were subsequently examined within the retained literature library and were not used as mandatory restrictions in the core searches.

# PubMed

("prostate cancer"[Title/Abstract] OR mCRPC[Title/Abstract] OR "metastatic castration-resistant prostate cancer"[Title/Abstract])
AND
("PSMA PET"[Title/Abstract] OR "PSMA-PET"[Title/Abstract] OR "prostate-specific membrane antigen PET"[Title/Abstract])
AND
("radioligand therapy"[Title/Abstract] OR "lutetium-177"[Title/Abstract] OR "177Lu-PSMA"[Title/Abstract] OR "Lu-177 PSMA"[Title/Abstract] OR theranostic*[Title/Abstract])

# Web of Science Core Collection

TS=(("prostate cancer" OR mCRPC OR "metastatic castration-resistant prostate cancer")
AND ("PSMA PET" OR "PSMA-PET" OR "prostate-specific membrane antigen PET")
AND ("radioligand therapy" OR "lutetium-177" OR "177Lu-PSMA" OR "Lu-177 PSMA" OR theranostic*))

# Search Yield

The searches yielded 374 PubMed records and 463 Web of Science records. After conservative deduplication, 578 unique records remained; 452 were retained as strongly relevant following title, abstract, and metadata screening
